# Robotic radical hysterectomy is superior to laparoscopic radical hysterectomy and open radical hysterectomy in the treatment of cervical cancer

**DOI:** 10.1371/journal.pone.0193033

**Published:** 2018-03-19

**Authors:** Yue-Mei Jin, Shan-Shan Liu, Jun Chen, Yan-Nan Chen, Chen-Chen Ren

**Affiliations:** 1 Department of Gynecology and Obstetrics, the Second Hospital of Jilin University, Changchun, P.R. China; 2 Department of Gynecology, The Third Affiliated Hospital of Zhengzhou University, Zhengzhou, P.R. China; The Wistar Institute, UNITED STATES

## Abstract

**Objective:**

Cervical cancer (CC) continues to be a global burden for women, with higher incidence and mortality rates reported annually. Many countries have witnessed a dramatic reduction in the prevalence of CC due to widely accessed robotic radical hysterectomy (RRH). This network meta-analysis aims to compare intraoperative and postoperative outcomes in way of RRH, laparoscopic radical hysterectomy (LTH) and open radical hysterectomy (ORH) in the treatment of early-stage CC.

**Methods:**

A comprehensive search of PubMed, Cochrane Library and EMBASE databases was performed from inception to June 2016. Clinical controlled trials (CCTs) of above three hysterectomies in the treatment of early-stage CC were included in this study. Direct and indirect evidence were incorporated for calculating values of weighted mean difference (WMD) or odds ratio (OR), and drawing the surface under the cumulative ranking curve (SUCRA).

**Results:**

Seventeen 17 CCTs were ultimately enrolled in this network meta-analysis. The network meta-analysis showed that patients treated by RRH and LRH had lower estimated blood loss compared to patients treated by ORH (WMD = -399.52, 95% CI = -600.64~-204.78; WMD = -277.86, 95%CI = -430.84 ~ -126.07, respectively). Patients treated by RRH and LRH had less hospital stay (days) than those by ORH (WMD = -3.49, 95% CI = -5.79~-1.24; WMD = -3.26, 95% CI = -5.04~-1.44, respectively). Compared with ORH, patients treated with RRH had lower postoperative complications (OR = 0.21, 95%CI = 0.08~0.65). Furthermore, the SUCRA value of three radical hysterectomies showed that patients receiving RRH illustrated better conditions on intraoperative blood loss, operation time, the number of resected lymph nodes, length of hospital stay and intraoperative and postoperative complications, while patients receiving ORH demonstrated relatively poorer conditions.

**Conclusion:**

The results of this meta-analysis confirmed that early-stage CC patients treated by RRH were superior to patients treated by LRH and ORH in intraoperative blood loss, length of hospital stay and intraoperative and postoperative complications, and RRH might be regarded as a safe and effective therapeutic procedure for the management of CC.

## Introduction

Cervical cancer (CC) is the 2^nd^ most common female cancer and is the leading cause of death in women [1]. Almost 85% of the CC burden happens in the developed regions and the incidence of CC in developing countries is high [2]. Lack of awareness, ineffective screening programs, being dwarfed by other health priorities and insufficient attention to women's health are factors contributing to the increasing incidence rate of CC [3]. CC can be detected at early stages and treated appropriately in developed countries like America, however, women in many countries face great challenges as various health care systems are unable to provide regular CC screening tests and treatment [4]. The greatest obstruction in the treatment for CC remains to be the delay in diagnosis and treatment [2]. One of the most frequented approaches for treating patients suffering from early-stage CC is open radical hysterectomy (ORH), contributing to short postoperative hospital stay and postoperative complication [5]. Consistently, radical hysterectomy (RH) is verified to be the main mode of treatment for patients with early-stage CC including International Federation of Gynecology and Obstetrics (FIGO) stages from I to II A [6]. Thus, the comparison of different methods in the treatment of CC is necessary in order to raise the quality of life for women plagued by CC.

The clinical stage and severity of CC determines the treatment plan, from surgery to a combination of radiation, chemotherapy and surgery in different situations [7]. Pelvic radiotherapy (RT) and intracavitary brachytherapy used to be the main treatment modes for patients with advanced CC, playing an important role in the treatment of CC [8]. One of the most aggressive surgical interventions in gynaecologic surgical oncology is pelvic exenteration, and remains to be the only potentially curative treatment of loco regional recurrence after CC [9]. Surgical management is a therapy option but it imposes radical pelvic surgery in order to achieve surgical resection with curative intent [10]. RH is one of the conventional surgical management regimens for early-stage CC, and is associated with postoperative morbidities like bladder dysfunction, sexual dysfunction and colorectal motility disorders [11]. Robotic radical hysterectomy (RRH) can be a dependable technology for the treatment of early-stage CC, and existing evidence suggests that patients undergoing RRH fare better than patients undergoing laparoscopic radical hysterectomy (LRH) in postoperative recovery, while patients treated by RRH and LRH show similar surgical outcomes and similar limitations in clinical practice [12]. RRH is proved to be a beneficial approach for early-stage CC patients, and LRH is a safer way compared to ORH owing to earlier recovery and fewer postoperative complications [5, 13]. Moreover, based on similar sample size and mass indexes, three RH showed different results of operation time, blood loss, transfusion rate, duration of stay in hospital, number of lymph nodes, positive margins and even post-operative infectious morbidity [14]. There are various effective regimens that are yet to be unfounded, and in order to provide important information to better assist the patients and physicians, reducing the morbidity and optimizing the treatment of this malignancy, we performed a network meta-analysis to compare different modes of hysterectomy in the treatment of early-stage CC to provide an optimal method of treatment based on literature.

## Materials and methods

### Search strategy

We retrieved PubMed, Cochrane Library and EMBASE databases to obtain literature relevant to this study, and relevant articles were also reviewed manually in case of the omission of any potentially relevant literature. The literature search was limited to the English language and ended in June 2016. The search terms included a combination of key words and free words as follows: (1) cervical cancer, cervical carcinoma, cervical neoplasms, uterine cervical cancer, neoplasm, uterine cervical, cervix neoplasms, cancer of the cervix, cervical cancers, uterine, neoplasms and cervical; (2) surgery, surgical procedures, operative, operative surgical procedures, and operative procedures; (3) hysterectomy and radical hysterectomy; (4) randomized, randomized controlled trial, placebo, double-blind method, controlled clinical trial (CCT), and cohort study.

### Inclusion and exclusion criteria

The inclusion criteria were as follows: (1) study design must be CCTs; (2) the interventions were RRH, LRH and ORH; (3) study subjects should be patients with early-stage CC aging from 15–85 years, body mass index (BMI, kg/m^2^) ranging was from 15–45, the type of histological cell should be squamous or adenocarcinoma, and patients suffering from early-stage CC were at the FIGO Stage I and II; (4) the outcomes of studies included estimated blood loss (ml), hospital stay (days), intraoperative complications, number of pelvic lymph nodes removed, operative time (min) and postoperative complications. The exclusion criteria were as follows: (1) patients previously undergone radiotherapy, chemotherapy and neoadjuvant therapy; (2) patients with celiaca; (3) pregnant or lactating patients; (4) studies lacking complete literature data; (5) non-CCTs; (6) duplications; (7) conference reports, meta-analysis and summaries; (8) non-English references.

### Data extraction and quality assessment

Two researchers independently carried out data extraction on the basis of a unified data collection form. Any dispute appearing during data extraction was resolved through discussion with multiple researchers. The quality of all included studies was assessed by researchers according to the Physiotherapy Evidence Database scale (PEDro) [15]. The total scores of PEDro were 11 points, score ≥ 4 points was regarded as high quality and score < 4 points was deemed as low quality [16]. The assessment consisted of a judgment of “yes,” “no,” or “unclear” for each domain to indicate a low, high, or unclear risk of bias, respectively. If one or no domain was deemed “unclear” or “no,” the study was classified as having a low risk of bias. If four or more domains were deemed “unclear” or “no,” the study was classified as having a high risk of bias. If two or three domains were deemed “unclear” or “no,” the study was regarded as having a moderate risk of bias [17]. The Review Manager 5 (RevMan 5.2.3, Cochrane Collaboration, Oxford, UK) statistical computing software was used to carry out quality assessment and investigation of publication bias.

### Statistical analysis

Firstly, traditional pairwise meta-analyses were performed for studies that compared different treatment arms directly. Our results reported the pooled estimates of odds ratios (ORs) or weighted mean difference (WMDs) and 95% confidence intervals (CIs). Heterogeneity among the studies was tested using the Chi-square test and I-square tests [18]. Random effect model was employed for the condition that the comparison results showed *I*^*2*^ > 50% and *P*_*h*_ < 0.05. Otherwise, fixed effect model was used for experiments. Secondly, R version 3.2.1 statistical computing software and network package were used to draw the network graphs, with each node representing different interventions, node size representing sample size, and the thickness of lines between the nodes indicating the number of included studies. Thirdly, Bayesian network meta-analyses were performed in order to compare different interventions with each other. Each analysis was performed based on the non-informative priors for effect sizes and precision. Convergence and lack of auto correlation were examined and confirmed after four chains and a 20,000-simulation burn-in phase; ultimately, direct probability statements were derived from an additional 50,000-simulation phase [19]. Furthermore, the node-splitting method was adopted in order to evaluate the consistency of the model, which separated evidence on a particular comparison into direct and indirect evidence [20]. To provide assistance in the interpretation of ORs, the surface under the cumulative ranking curve (SUCRA) was used in order to calculate the probability of each intervention being the most effective diagnostic method based on a Bayesian approach using probability values, and the larger the SUCRA value, the better the rank of the intervention [21, 22]. Cluster analyses SUCRA values were conducted in order to group and rank the treatments according to their similarity with respect to two outcomes [21]. All computations were carried out by R (V.3.2.1) package gemtc (V.0.6), along with the Markov Chain Monte Carlo engine Open BUGS (V.3.4.0).

## Results

### Baseline characteristics of included studies

**Fig 1:**
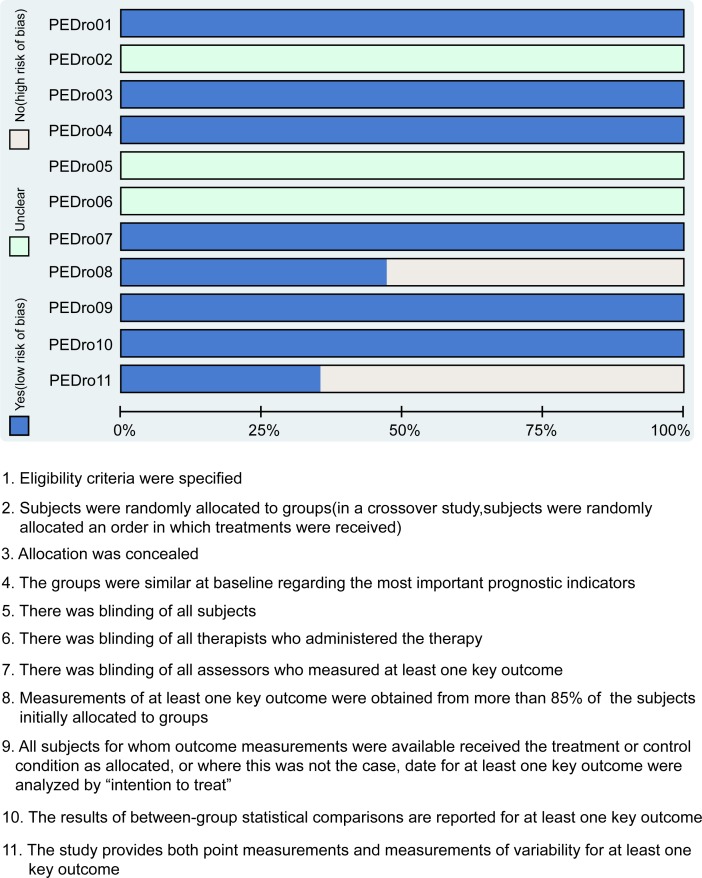
Quality assessment based on PEDro scale of clinical controlled trials included in the network meta-analysis. Note: PEDro = Physiotherapy Evidence Database.

A total of 2,614 studies relevant to this study initially retrieved. After excluding duplicate studies (n = 26), letters, reviews or meta-analysis (n = 759), non-human studies (n = 231) and 326 non-English studies (n = 326), a total of 1,272 studies were evaluated for eligibility by full-text review. After full-text review, non-cohort literatures (n = 260), unrelated CC studies (n = 630), 362 irrelevant with surgical treatment studies (n = 362) and studies lacking data or with incomplete data (n = 2) studies were ruled out. Finally, 17 CCTs were incorporated in this network meta-analysis. [5, 13, 23–37] (S1 Fig). The included studies were published between 2007 and 2015. These studies included 2,100 early-stage CC patients treated with radical hysterectomies and a majority of the patients underwent ORH and LRH. Among the 17 included studies, 11 CCTs were performed in Caucasians, and 6 CCTs were performed in Asians. Furthermore, 14 included studies were two-arm trials, and 3 studies were three-arm trials. The baseline characteristics of the included studies were shown in S1 Table and the PEDro score was shown in Fig 1.

### Pairwise meta-analysis of intraoperative and postoperative outcomes of RRH, LRH and ORH on early-stage CC

We conducted a pairwise meta-analysis to compare the intraoperative and postoperative outcomes of three radical hysterectomies on early-stage CC. The results revealed that patients opting for RRH and LRH exhibited lower estimated blood loss (ml) compared to the patients opting for ORH (WMD = -228.68, 95% CI = -281.56~-175.81; WMD = -515.43, 95% CI = -767.92~-262.94, respectively). Compared with ORH, patients undergoing LRH had relatively longer operative time periods (min) (WMD = 20.77, 95% CI = 3.35~38.18). Patients undergone RRH demonstrated less number of pelvic lymph nodes removed than those received ORH (WMD = -2.25, 95% CI = -4.03~-0.48). However, there were no significant differences in three radical hysterectomies. Patients received RRH and LRH had shorter hospital stays (days) than those received ORH (WMD = -3.05, 95% CI = -4.75~-1.36; WMD = -3.41, 95% CI = -4.93~-1.90, respectively). RRH patients showed a lower incidence of postoperative complications compared to LRH patients (OR = 0.42, 95% CI = 0.20~0.87), while LRH patients had a lower incidence of postoperative complications compared to ORH patients (OR = 0.53, 95% CI = 0.37~0.75), which indicated that patients received RRH had lower incidence of postoperative complications in early-stage CC (Table 1 and S2–S4 Figs).

**Table 1:** Pairwise meta-analysis in terms of six endpoints.

Included studies	Comparisons	Pairwise meta-analysis			
		WMD/OR (95%CI)	*I*^2^	*P*_*h*_	Model
**Estimated blood loss(ml)**					
5 studies	RRH vs. LRH	-40.39(-117.75~35.97)	96%	<0.01	Random effect
5 studies	RRH vs. ORH	**-228.68(-281.56~-175.81)**	96%	<0.01	Random effect
10 studies	LRH vs. ORH	**-515.43(-767.92~-262.94)**	97%	<0.01	Random effect
**Operative time(min)**					
5 studies	RRH vs. LRH	-8.24(-61.56~45.07)	97%	<0.01	Random effect
5 studies	RRH vs. ORH	25.25(-28.48~78.98)	97%	<0.01	Random effect
12 studies	LRH vs. ORH	**20.77(3.35~38.18)**	98%	<0.01	Random effect
**Number of pelvic lymph nodes removed**					
4 studies	RRH vs. LRH	-0.53(-1.90~0.85)	55%	0.08	Fixed effect
4 studies	RRH vs. ORH	**-2.25(-4.03~-0.48)**	32%	0.22	Fixed effect
9 studies	LRH vs. ORH	-0.83(-2.86~1.20)	86%	<0.01	Random effect
**Intraoperative complications**					
3 studies	RRH vs. LRH	0.83(0.16~4.34)	63%	0.07	Fixed effect
3 studies	RRH vs. ORH	0.51(0.13~2.02)	0%	0.77	Fixed effect
7 studies	LRH vs. ORH	1.22(0.73~2.04)	0%	0.76	Fixed effect
**Hospital stay(days)**					
4 studies	RRH vs. LRH	-1.01(-2.82~0.80)	92%	<0.01	Random effect
4 studies	RRH vs. ORH	**-3.05(-4.75~-1.36)**	94%	<0.01	Random effect
9 studies	LRH vs. ORH	**-3.41(-4.93~-1.90)**	98%	<0.01	Random effect
**Postoperative complications**					
2 studies	RRH vs. LRH	**0.42(0.20~0.87)**	0%	0.34	Fixed effect
3 studies	RRH vs. ORH	0.31(0.05~2.02)	72%	0.03	Random effect
7 studies	LRH vs. ORH	**0.53(0.38~0.75)**	8%	0.37	Fixed effect

Notes: WMD, weighted mean difference; OR, odds radio; CI, confidence intervals; RRH, robotic radical hysterectomy; LRH, laparoscopic radical hysterectomy; ORH, open radical hysterectomy. Estimated blood loss, hospital stay, number of pelvic lymph nodes removed and operative time are stated as WMD values, while intraoperative complications and postoperative complications are stated as OR values.

### Network evidence results suggesting more patients received RRH and LRH while less received ORH in the treatment of early-stage CC

**Fig 2:**
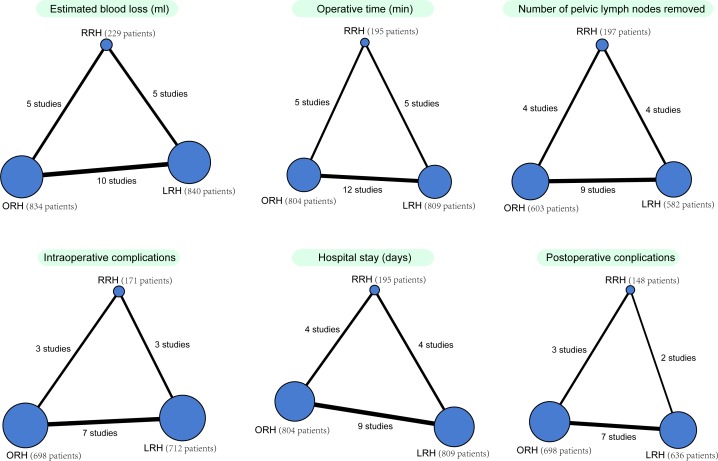
Network evidence plots of estimated blood loss (ml), operative time (min), number of pelvic lymph nodes removed, intraoperative complications, hospital stay (days) and postoperative complications of RRH, LRH and ORH in the treatment of early-stage CC. Note: RRH, robotic radical hysterectomy; LRH, laparoscopic radical hysterectomy; ORH, open radical hysterectomy; CC cervical cancer.

The following three radical hysterectomies were included in this study: RRH, LRH and ORH. We found that a large number of patients underwent ORH and LRH, while the least number of patients underwent RRH (Fig 2).

### The main results of network meta-analysis of intraoperative and postoperative outcomes of RRH, LRH and ORH in the treatment of early-stage CC

**Fig 3:**
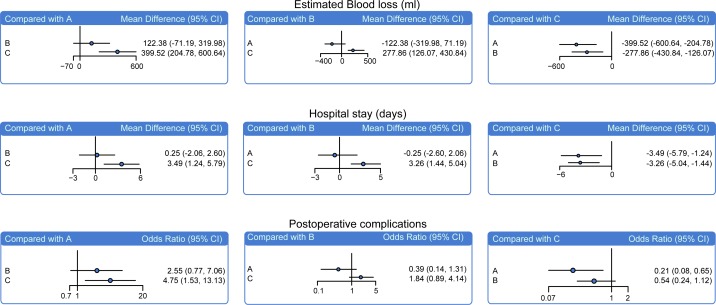
Relative relationship forest plots of estimated blood loss (ml), hospital stay (days) and postoperative complications of RRH, LRH and ORH in the treatment of early-stage CC. Note: A = RRH (robotic radical hysterectomy); B = LRH (laparoscopic radical hysterectomy); C = ORH (open radical hysterectomy).

The network meta-analysis showed that patients treated by RRH and LRH had lower estimated blood loss than those treated by ORH (WMD = -399.52, 95% CI = -600.64~-204.78; WMD = -277.86, 95% CI = -430.84~-126.07, respectively). Patients treated by RRH and LRH had shorter me. hospital stays than patients treated by ORH (WMD = -3.49, 95% CI = -5.79~-1.24; WMD = -3.26, 95% CI = -5.04~-1.44, respectively). Compared with ORH patients, RRH patients exhibited lower postoperative complications (OR = 0.21, 95% CI = 0.08~0.65). However, no significant differences in terms of operative time (min), number of pelvic lymph nodes removed and intraoperative complications were found among the three radical hysterectomies (Fig 3, S5 Fig and Table 2).

**Table 2:** Weighted mean difference or odds ratio (95% confidence intervals) of three treatment modalities of six endpoint outcomes.

WMD/OR (95%CI)		
**Estimated blood loss (ml)**		
**RRH**	122.38 (-71.19, 319.98)	**399.52 (204.78, 600.64)**
-122.38 (-319.98, 71.19)	**LRH**	**277.86 (126.07, 430.84)**
**-399.52 (-600.64, -204.78)**	**-277.86 (-430.84, -126.07)**	**ORH**
**Operative time(min)**		
**RRH**	-7.29 (-54.25, 40.40)	-24.24 (-69.02, 24.44)
7.29 (-40.40, 54.25)	**LRH**	-17.09 (-49.80, 18.65)
24.24 (-24.44, 69.02)	17.09 (-18.65, 49.80)	**ORH**
**Number of pelvic lymph nodes removed**		
**RRH**	1.34 (-1.89, 4.44)	2.17 (-1.08, 5.49)
-1.34 (-4.44, 1.89)	**LRH**	0.84 (-1.47, 3.23)
-2.17 (-5.49, 1.08)	-0.84 (-3.23, 1.47)	**ORH**
**Intraoperative complications**		
**RRH**	1.87 (0.29, 11.01)	1.74 (0.28, 11.17)
0.53 (0.09, 3.48)	**LRH**	0.90 (0.30, 3.10)
0.58 (0.09, 3.55)	1.11 (0.32, 3.36)	**ORH**
**Hospital stay(days)**		
**RRH**	0.25 (-2.06, 2.60)	**3.49 (1.24, 5.79)**
-0.25 (-2.60, 2.06)	**LRH**	**3.26 (1.44, 5.04)**
**-3.49 (-5.79, -1.24)**	**-3.26 (-5.04, -1.44)**	**ORH**
**Postoperative complications**		
**RRH**	2.55 (0.77, 7.06)	**4.75 (1.53, 13.13)**
0.39 (0.14, 1.31)	**LRH**	1.84 (0.89, 4.14)
**0.21 (0.08, 0.65)**	0.54 (0.24, 1.12)	**ORH**

**Notes:** WMD, weighted mean difference; OR, odds radio; CI, confidence intervals; RRH, robotic radical hysterectomy; LRH, laparoscopic radical hysterectomy; ORH, open radical hysterectomy. Estimated blood loss, hospital stay, number of pelvic lymph nodes removed and operative time are stated as WMD values, while intraoperative complications and postoperative complications are stated as OR values.

### Inconsistency test of network meta-analysis of intraoperative and postoperative outcomes of RRH, LRH and ORH in the treatment of early-stage CC

The node-splitting method was used in order to test for inconsistencies for the six outcomes, and found that was consistent with the direct evidence and the indirect evidence so that we should use consistency model (all *P* > 0.05) (Table 3).

**Table 3:** WMD/OR values and *P* values of direct and indirect pairwise comparisons of three treatment modalities under six endpoint outcomes.

Pairwise comparisons	Direct WMD/OR values						Indirect WMD/OR values						*P* values					
	E	O	N	I	H	P	E	O	N	I	H	P	E	O	N	I	H	P
B vs. A	38	7.7	0.8	1	1	2.2	130	-56	4.1	4.1	-1.5	3.9	0.311	0.176	0.459	0.419	0.338	0.658
C vs. A	520	-25	2.2	2.1	3.1	4.2	140	-39	2.8	0.2	3.8	5.5	0.083	0.810	0.861	0.240	0.771	0.848
C vs. B	270	-19	0.9	0.8	3.4	1.8	350	-40	2.5	18	2.2	2.5	0.763	0.638	0.705	0.151	0.633	0.821

Notes: WMD = weighted mean difference; OR = odds radio; E = Estimated blood loss; H = Hospital stay; I = Intraoperative complications; N = Number of pelvic lymph nodes removed; O = Operative time; P = Postoperative complications; A = RRH (robotic radical hysterectomy); B = LRH (laparoscopic radical hysterectomy); C = ORH (open radical hysterectomy); Estimated blood loss, hospital stay, number of pelvic lymph nodes removed and operative time are stated as WMD values, while intraoperative complications and postoperative complications are stated as OR values.

### RRH has the highest SUCRA values in estimated blood loss (ml), operative time (min), number of pelvic lymph nodes removed, intraoperative complications, hospital stay (days) and postoperative complications in the treatment of early-stage CC

As shown in Table 4, the SUCRA value of cumulative probability sorting of intraoperative and postoperative of three radical hysterectomies on early-stage CC showed that RRH had the highest SUCRA values of estimated blood loss (ml), operative time (min), number of pelvic lymph nodes removed, intraoperative complications, hospital stay (days) and postoperative complications (96.67%, 82.67%, 90.67%, 82.33%, 86.33%, and 98.33%). ORH demonstrated the lowest SUCRA values of estimated blood loss (ml), operative time (min), number of pelvic lymph nodes removed, hospital stay (days) and postoperative complications (33.33%, 43.67%, 43.33%, 33.33%, and 35.00%). LRH demonstrated the lowest SUCRA value of intraoperative complications (55.33%). In conclusion, patients receiving RRH proved less estimated blood loss (ml), short operative time (min) and hospital stay (days), more number of pelvic lymph nodes removed and less intraoperative and postoperative complications among three radical hysterectomies.

**Table 4:** SUCRA values of three treatment modalities under six endpoint outcomes.

Treatments	SUCRA values (%)					
	Estimated blood loss	Operative time	Number of pelvic lymph nodes removed	Intraoperative complications	Hospital stay	Postoperative complications
**A**	**96.67**	**82.67**	**90.67**	**82.33**	**86.33**	**98.33**
**B**	70.00	74.00	66.00	55.33	80.33	67.00
**C**	33.33	43.67	43.33	62.00	33.33	35.00

Notes: A = RRH (robotic radical hysterectomy); B = LRH (laparoscopic radical hysterectomy); C = ORH (open radical hysterectomy).

### Based on cluster analysis results, RRH had better intraoperative and postoperative clinical outcomes in the treatment of early-stage CC

**Fig 4:**
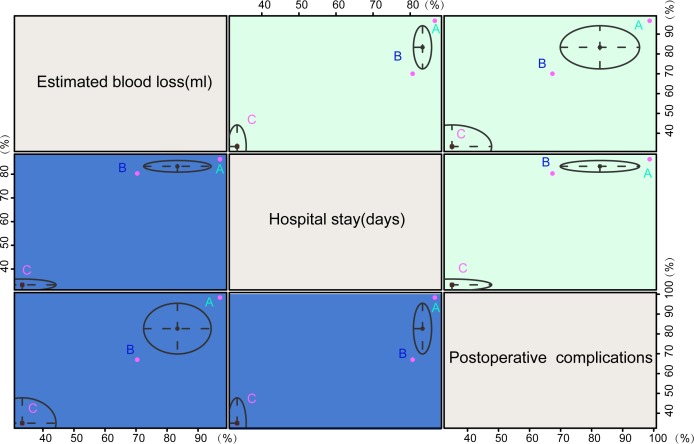
Cluster analysis diagram of estimated blood loss (ml), hospital stay (days) and postoperative complications of RRH, LRH and ORH in the treatment of early-stage CC. Note: A = RRH (robotic radical hysterectomy); B = LRH (laparoscopic radical hysterectomy); C = ORH (open radical hysterectomy).

Cluster analysis of SUCRA values based on estimated blood loss (ml), operative time (min), number of pelvic lymph nodes removed, intraoperative complications, hospital stay (days) and postoperative complications showed that patients treated by RRH showed better intraoperative and postoperative clinical outcomes in the treatment of early-stage CC, while patients treated by ORH had the worst conditions (Fig 4).

### Assessment of publication bias of intraoperative and postoperative outcomes of RRH, LRH and ORH in the treatment of early-stage CC

**Fig 5:**
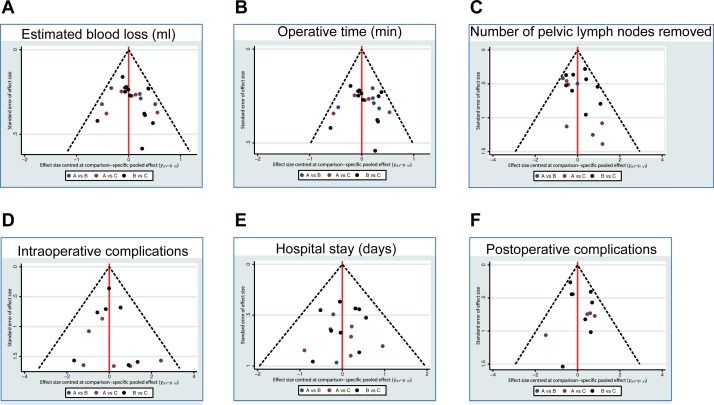
Funnel plot of estimated blood loss (ml), operative time (min), number of pelvic lymph nodes removed, intraoperative complications, hospital stay (days) and postoperative complications of RRH, LRH and ORH in the treatment of early-stage CC. Note: A = RRH (robotic radical hysterectomy); B = LRH (laparoscopic radical hysterectomy); C = ORH (open radical hysterectomy).

The results of assessment of publication bias showed symmetrical distribution, indicating no small sample effect or publication bias in this network meta-analysis All the scattered points were of hypodispersion in the funnel, and red lines were symmetrical on both sides, indicating that the bias of reference applied in our study was small (Fig 5).

## Discussion

In this study, we evaluated three different approaches for hysterectomies in the treatment of CC by summarizing clinical data in a pairwise meta-analysis. Our clinical data supports previous findings and suggests that RRH and LRH have better intraoperative and postoperative outcomes compared to ORH in the treatment of CC. Currently, an increasing number of gynecologists opt for the RRH approach in order to decrease postoperative morbidity [12]. Robot assisted surgery allows greater visualization of the instrument by means of binocular vision, using seven degrees of freedom of the instrument with greater flexibility, and the motion of the damping control is more accurate in 2005. [35]. LRH is performed routinely all around the world, due to the advances in minimally invasive surgery [12]. A recent meta-analysis showed that a comparison of RRH and PRH was not practicable as a result of insufficiency in studies that assessed appropriate “radical” hysterectomy merely for uterine CC [38]. Many clinicians believe that the RRH is associated with a lower incidence of postoperative morbidity compared to the traditional relative humidity, with similar clinical efficacy and safety [12].

The network meta-analysis showed that patients treated by RRH and LRH had lower estimated blood loss compared to patients undergoing ORH. Patients treated by RRH and LRH had shorter hospital stays than ORH. Compared with ORH, patients undergoing RRH treatment demonstrated lower postoperative complications. A previous meta-analysis showed that LRH and RRH were similar in terms of operating time, length of hospital stay, and number of pelvic lymph nodes resected, and RRH presented an overwhelming advantage and less blood loss against LRH with respect to complications [12]. Compared with ORH, RRH indicated lower blood loss and shorter length of hospital stays [35]. It is quite difficult to draw comprehensive conclusions from different studies about operative time, blood loss, and number of lymph nodes, however, the overall consensus is that a minimally invasive technique seems to be the best laparoscopic radical hysterectomy to treat CC [33]. Soliman *et al* reported that RRH is associated with shortened hospital stay and reduced blood loss, nevertheless, the LRH and LRH all showed longer operation time than the laparotomy [27].

The results of the cluster analysis showed that the SUCRA value of RRH is higher than that of LRH and ORH as seen in Fig 4. The SUCRA value of RRH suggested relatively better clinical outcomes of intraoperative and postoperative complications in early-stage CC. Compared with the other two surgical groups, the robotic group showed postoperative parameters that reduced postoperative and 24-hour pain scores, shortened the length of hospital stay, and reduced the time to full diet resumption [27]. The results of the study proved that comparable surgical outcomes of patients receiving RRH of traditional laparoscopic approach in the treatment of early-stage CC, with lower intraoperative blood loss and early complication rates [24]. Chen CH *et al* reported that robotic surgery is verified to have a lower proficiency plateau and relatively shorter learning curve than traditional approaches [27]. Blood loss, rate of blood loss and length of hospital stay are similar for laparoscopy and robotics, and are significantly reduced as compared with laparotomy [39]. The data suggested that robotic surgery is a workable and potentially optimal option to treat CC with favorable short-term surgical outcomes [27].

However, significant differences for the number of RRH, LRH and ORH on the direct comparison of various interventions and the sample size of each intervention our present network meta-analysis had also limitation and advantage: (1) the sample size of each intervention, which might influence the overall results of the study; (2) in this research, our study showed the significant difference of RRH, LRH and ORH on hysterectomy in the treatment of early-stage CC; (3) due to lack of sufficient summarized studies to evaluate the long-term clinical outcomes between different treatment methods, we only focused on the comparisons of the short-term clinical outcomes using network meta-analysis.

## Conclusion

In conclusion, these results of our meta-analysis indicate that patients with early-stage CC treated by RRH had better clinical outcomes of intraoperative blood loss, length of hospital stay and intraoperative and postoperative complications than LRH and ORH, which has a certain guiding significance for the clinical use and treatment of early-stage CC.

## Supporting information

S1 PRISMA Checklist The index for this text. (DOC)

S1 Table The baseline characteristics for included studies. (DOCX)

S1 Fig Flow chart showing literature search and study selection. A sum of 17 clinical controlled trials containing 2,100 CC patients included in the network meta-analysis.
(EPS)

S2 Fig The traditional forest plots of estimated blood loss (ml) and operative time (min) of RRH, LRH and ORH in the treatment of early-stage CC. Note: A = RRH (robotic radical hysterectomy); B = LRH (laparoscopic radical hysterectomy); C = ORH (open radical hysterectomy).
(EPS)

S3 Fig The traditional forest plots of number of pelvic lymph nodes removed and intraoperative complications of RRH, LRH and ORH in the treatment of early-stage CC. Note: A = RRH (robotic radical hysterectomy); B = LRH (laparoscopic radical hysterectomy); C = ORH (open radical hysterectomy).
(EPS)

S4 Fig The traditional forest plots of hospital stay (days) and postoperative complications of RRH, LRH and ORH in the treatment of early-stage CC. Note: A = RRH (robotic radical hysterectomy); B = LRH (laparoscopic radical hysterectomy); C = ORH (open radical hysterectomy).
(EPS)

S5 Fig Relative relationship forest plots of operative time (min), number of pelvic lymph nodes removed and intraoperative complications of RRH, LRH and ORH in the treatment of early-stage CC. Note: A = RRH (robotic radical hysterectomy); B = LRH (laparoscopic radical hysterectomy); C = ORH (open radical hysterectomy).
(EPS)
