# Supplementary material for: Robotic radical hysterectomy is superior to laparoscopic radical hysterectomy and open radical hysterectomy in the treatment of cervical cancer
Source: PLoS One. 2018 Mar 19;13(3):e0193033. doi: 10.1371/journal.pone.0193033 (PMC5858845; doi:10.1371/journal.pone.0193033)
Supplement: S1 Table — (DOCX) [file pone.0193033.s002.docx]

**S1 Table. The baseline characteristics for included studies.**

| **First author** | **Year** | **Country** | **FIGO Stage** | **Interventions** | | | **Total** | **Sample size** | | | **Age (years)** | | | **BMI (kg/m²)** | | |
| --- | --- | --- | --- | --- | --- | --- | --- | --- | --- | --- | --- | --- | --- | --- | --- | --- |
|  |  |  |  | **T1** | **T2** | **T3** |  | **T1** | **T2** | **T3** | **T1** | **T2** | **T3** | **T1** | **T2** | **T3** |
| Ditto A | 2015 | Italy | IA2-IB1 | B | C | - | 120 | 60 | 60 | - | 46(29-79) | 45.5(15-78) | - | 24.3±2.9 | 24.0±4.3 | - |
| Yim GW | 2014 | Korea | IA1-IIA2 | A | B | - | 102 | 60 | 42 | - | 46.3±9.9 | 49.8±11.4 | - | 22.5(18.2-38.6) | 22.7(15.8-33.3) | - |
| Toptas T | 2014 | Turkey | IA2-IB2 | B | C | - | 68 | 22 | 46 | - | 46.5(40-57) | 50(46–58) | - | NR | NR | - |
| Kong TW | 2014 | Korea | IB-IIA | B | C | - | 88 | 40 | 48 | - | 45.0±10.6 | 48.0±11.0 | - | 22.3±2.9 | 23.4±3.3 | - |
| Chen CH | 2014 | China | IA-IIB | A | B | C | 100 | 24 | 32 | 44 | 53.7±15.3 | 51.2±11.9 | 51.9±11.3 | 24.4±4.9 | 23.2±3.4 | 24.9±4.6 |
| Park JY | 2013 | Korea | IB2-IIA2 | B | C | - | 303 | 115 | 188 | - | 48.5(25-77) | 48.1(25-84) | - | 23.1(15.62-34.80) | 23.7(17.63-34.75) | - |
| Campos LS | 2013 | Brazil | IA-IB | B | C | - | 30 | 16 | 14 | - | 36.19±9.78 | 39.64±6.23 | - | NR | NR | - |
| Nam JH | 2012 | Korea | IA2-IIA | B | C | - | 526 | 263 | 263 | - | NR | NR | - | NR | NR | - |
| Tinelli R | 2011 | Italy | IA1- IIA | A | B | - | 99 | 23 | 76 | - | 43.1±8.9 | 41.9±7.1 | - | 28±4 | 29±3 | - |
| Taylor SE | 2011 | America | IA2-IB1 | B | C | - | 27 | 9 | 18 | - | 41.4(31-60) | 41.1(25-61) | - | 26.3(20.6-36.1) | 26.9(17-38.3) | - |
| Soliman PT | 2011 | America | 1A1-IIA | A | B | C | 95 | 34 | 31 | 30 | 52.1(27.9-75.9) | 44.2(23.55-64.9) | 48.1(25.5-82.2) | 26.9 | 29.5 | 26.2 |
| Malzoni M | 2009 | Italy | IA1-IB1 | B | C | - | 127 | 65 | 62 | - | 40.5±7.7 | 42.7±8.6 | - | 26(19-35) | 29(19-35) | - |
| Maggioni A | 2009 | America | IA2-IIA | A | C | - | 80 | 40 | 40 | - | 44.1±9.1 | 49.8±14.1 | - | 24.1±5.5 | 23.6±5.0 | - |
| Estape R | 2009 | America | IA2-IB2 | A | B | C | 63 | 32 | 17 | 14 | 55.0±12.7 | 52.8±14.2 | 42.0±12.0 | 29.7±3.2 | 28.1±4.8 | 29.5±6.4 |
| Ko EM | 2008 | America | I-II | A | C | - | 48 | 16 | 32 | - | 42.3±7.9 | 41.7±8.1 | - | 27.6±6.4 | 26.6±5.9 | - |
| Li G | 2007 | China | IB-IIA | B | C | - | 125 | 90 | 35 | - | 42±9 | 44±11 | - | NR | NR | - |
| Frumovitz M | 2007 | America | IA2-IB2 | B | C | - | 89 | 35 | 54 | - | 40.8(28.4-63.4) | 42.5(27.3-68.3) | - | 28.2(17.4-46.4) | 28.1(18.4-40.8) | - |

Note: FIGO= the International Federation of Gynecology and Obstetrics; T = treatment; M = male; F = female; NR = not report; BMI = body mass index; A = RRH (robotic radical hysterectomy); B = LRH (laparoscopic radical hysterectomy); C = ORH (open radical hysterectomy).
